# Follistatin-like 1 promotes proliferation of matured human hypoxic iPSC-cardiomyocytes and is secreted by cardiac fibroblasts

**DOI:** 10.1016/j.omtm.2022.02.005

**Published:** 2022-02-23

**Authors:** Marijn C. Peters, Sofia Di Martino, Thomas Boelens, Jiabin Qin, Alain van Mil, Pieter A. Doevendans, Steven A.J. Chamuleau, Joost P.G. Sluijter, Klaus Neef

**Affiliations:** 1 Department of Cardiology, Laboratory of Experimental Cardiology, Regenerative Medicine Centre Utrecht, University Medical Centre Utrecht, University Utrecht, 3584 CX Utrecht, the Netherlands; 2 Department of Cardiology, Amsterdam Medical Centre, 1105 AZ Amsterdam, the Netherlands

**Keywords:** Follistatin-like 1, iPSC-cardiomyocytes, ischemic heart disease, hypoxia, fibroblasts

## Abstract

The human heart has limited regenerative capacity. Therefore, patients often progress to heart failure after ischemic injury, despite advances in reperfusion therapies generally decreasing mortality. Depending on its glycosylation state, Follistatin-like 1 (FSTL1) has been shown to increase cardiomyocyte (CM) proliferation, decrease CM apoptosis, and prevent cardiac rupture in animal models of ischemic heart disease. To explore its therapeutic potential, we used a human *in vitro* model of cardiac ischemic injury with human induced pluripotent stem cell-derived CMs (iPSC-CMs) and assessed regenerative effects of two differently glycosylated variants of human FSTL1. Furthermore, we investigated the FSTL1-mediated interplay between human cardiac fibroblasts (cFBs) and iPSC-CMs in hypoxia. Both FSTL1 variants increased viability, while only hypo-glycosylated FSTL1 increased CM proliferation post-hypoxia. Human fetal cardiac fibroblasts (fcFBs) expressed and secreted FSTL1 under normoxic conditions, while FSTL1 secretion increased by iPSC-cFBs upon hypoxia but decreased in iPSC-CMs. Co-culture of iPSC-CMs and cFBs increased FSTL1 secretion compared with cFB mono-culture. Taken together, we confirm that FSTL1 induces iPSC-CM proliferation in a human cardiac *in vitro* hypoxia damage model. Furthermore, we show hypoxia-related FSTL1 secretion by human cFBs and indications for FSTL1-mediated intercellular communication between cardiac cell types in response to hypoxic conditions.

## Graphical Abstract

**Figure:**
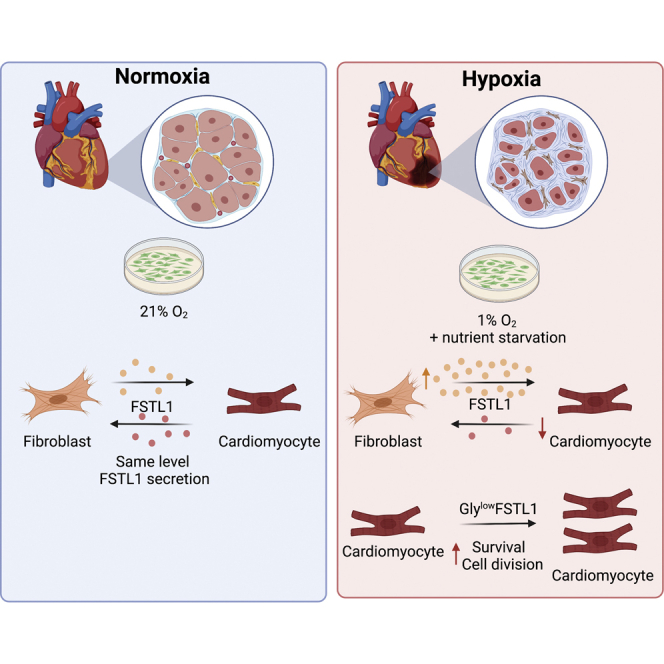

## Teaser

Peters et al. report that hypo-glycosylated Follistatin-like 1 (FSTL1) induces proliferation of metabolically matured human iPSC-cardiomyocytes (CMs). Both iPSC-CMs and cardiac fibroblasts secrete FSTL1; hypoxia decreases CM and increases fibroblast FSTL1 secretion. Co-culture of iPSC-CMs and fibroblasts promotes FSTL1 secretion, indicating a role of FSTL1 in hypoxia-responsive intercellular communication.

## Introduction

The human heart has limited regenerative capacity and repairs itself poorly after injury. After ischemic injury, patients often progress to heart failure despite the decrease in direct mortality by reperfusion therapies.^1^^,^^2^ In contrast to the adult human heart, neonatal vertebrate and invertebrate hearts can substantially regenerate from injury or disease via induced proliferation of cardiomyocytes (CMs) *in situ.*^3^^,^^4^ As damaged adult CMs in the human heart show up-regulation of genes involved in heart development and invertebrate heart regeneration, a rudimentary cardiac regeneration mechanism seems to exist, yet it is inefficient to cope with massive CM damage or loss (e.g., inflicted by ischemic insult from myocardial infarction).^5^, ^6^, ^7^, ^8^, ^9^ Thus, stimulation of the CM cell cycle to potentially re-establish regenerative capacity in the adult human heart has become a key focus in the field of advanced cardiac therapies. Induction of CM proliferation in regenerating hearts has been linked to paracrine signaling, often attributed to secretion from the epicardium.^10^, ^11^, ^12^, ^13^, ^14^ Furthermore, efficient cardiac regeneration has been shown to be accompanied by revascularization induced by epicardial derived endothelial and smooth muscle cells.^15^ These cellular responses to tissue damage rely on intercellular communication via soluble paracrine factors^16^ and the close proximity of endothelial cells, cardiac fibroblasts (cFBs), and CMs.^17^^,^^18^ Changes in the cellular composition after an ischemic insult (i.e., increased frequency of cFBs and decreased frequency of CMs and endothelial cells) alter paracrine signaling and cross-talk between cardiac cells and thus effects damage response.^16^^,^^19^ The epicardium has been identified to be instrumental in mediating cardiac regeneration initially in the zebrafish heart, and epicardially secreted glycoprotein Follistatin like-1 (FSTL1) has been confirmed to act as myogenic cardiokine in murine and porcine cardiac injury models.^20^ Furthermore, cardioprotective and angiogenic effects of FSTL1 have been reported.^21^, ^22^, ^23^, ^24^, ^25^, ^26^, ^27^, ^28^ FSTL1 is a 308 amino acid glycoprotein of the SPARC protein family, with three N-glycosylation sites,^27^^,^^29^, ^30^, ^31^ with one (N180) being critically linked to the role of FSTL1 in cardiac regeneration.^31^ Additionally, an increase in secretion of FSTL1 with higher molecular weight, likely due to protein glycosylation, could be observed in mouse serum and myocardium after myocardial infarction.^20^^,^^28^ All this strongly suggests that regulation of FSTL1 expression and glycosylation after myocardial infarction could guide approaches on exploiting its regenerative potential for the heart. Besides its role in CM renewal, the expression of FSTL1 by cFBs in the infarct area was found to be essential to prevent cardiac rupture.^32^^,^^33^

As indicated, so far animal studies have shed some light on FSTL1 as a potential therapeutic agent to induce cardiac regeneration by stimulating both CM proliferation, protection from apoptosis, revascularization, and stabilizing the infarct scar. However, these findings have yet to be confirmed in a human setting. The development of CMs derived from human induced pluripotent stem cells (iPSC-CMs) has become a new promising way to model the complex cellular physiology of human cardiac cells, while taking into account the usually relatively immature phenotype of iPSC-CMs.^34^ Using methods to stimulate metabolic maturation of iPSC-CMs and stimulate oxidative phosphorylation-based energy metabolism^35^ to improve their susceptibility to hypoxic damage and thus their applicability to model ischemic heart disease.

In this study, we sought to assess the cardiac regenerative capacity of FSTL1, taking into account the glycosylation state, using a human iPSC-CM-based *in vitro* hypoxia model, and exploring a potential role of cFBs.

## Results

### Hypo- and hyperglycosylated FSTL1 variants exert cardioprotective effects

**Figure 1:**
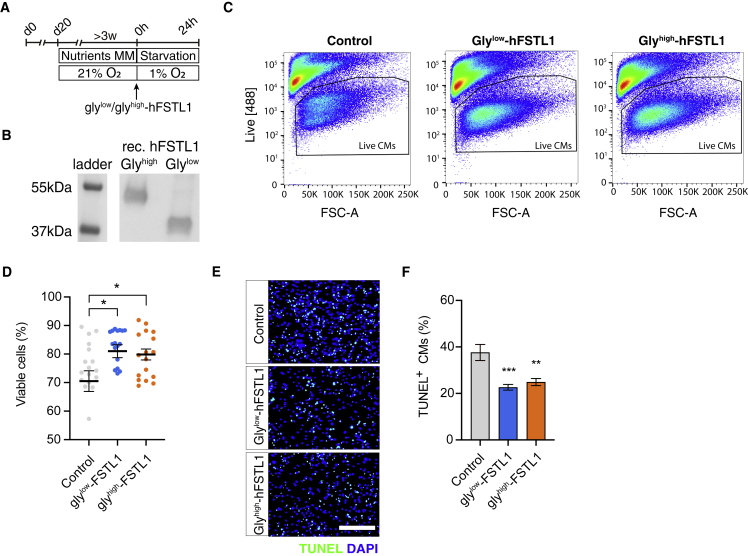
FSTL1 increases survival of hypoxic matured iPSC cardiomyocytes (A) Schematic representation of experimental setup. (B) Western blot analysis of gly^low^-FSTL1 and gly^high^-FSTL1. (C) Flow cytometry analysis of live dead staining. (D) Quantification of (C). (E) Microscopic images of TUNEL-stained iPSC-CM. (F) Quantification of (E) (n = 3 biological replicates). Data was analyzed using one-way ANOVA and Dunnett multiple-comparisons test. ∗p < 0.05, ∗∗p < 0.01, and ∗∗∗p < 0.001. Scale bar: 200 μm. Data are presented as mean ± SEM. To see this figure in color, go online.

We analyzed cardioprotective effects of FSTL1 and the effect of FSTL1 glycosylation by treating CMs with bacterially produced human recombinant FSTL1 (low-glycosylated [gly^low^-FSTL1]) or mammalian produced human recombinant FSTL1 (high-glycosylated [gly^high^-FSTL1]) at the onset of 24 h hypoxia (Figure 1A). The increased molecular weight of gly^high^-FSTL1 was confirmed using western blot (50 vs. 37 kDa [gly^low^-FSTL1]) (Figure 1B). Flow cytometric analysis showed a significant increase in viability after treatment with either glycosylation variant compared with control (from 70% ± 3.6% [control] to 81% ± 2.3% [gly^low^-FSTL1] or 79.4% ± 1.9% [gly^high^-FSTL1], p < 0.05; Figures 1C and 1D). Furthermore, both glycosylation variants of FSTL1 decreased the number of TUNEL^+^ iPSC-CMs (from 37.6 ± 3.5 [control] to 22.6 ± 1.2 [gly^low^-FSTL1] or 24.9 ± 1.5 [gly^high^-FSTL1], p < 0.01; Figures 1E and 1F).

### Hypo-glycosylated FSTL1 increases proliferation of hypoxic mature iPSC-CMs

**Figure 2:**
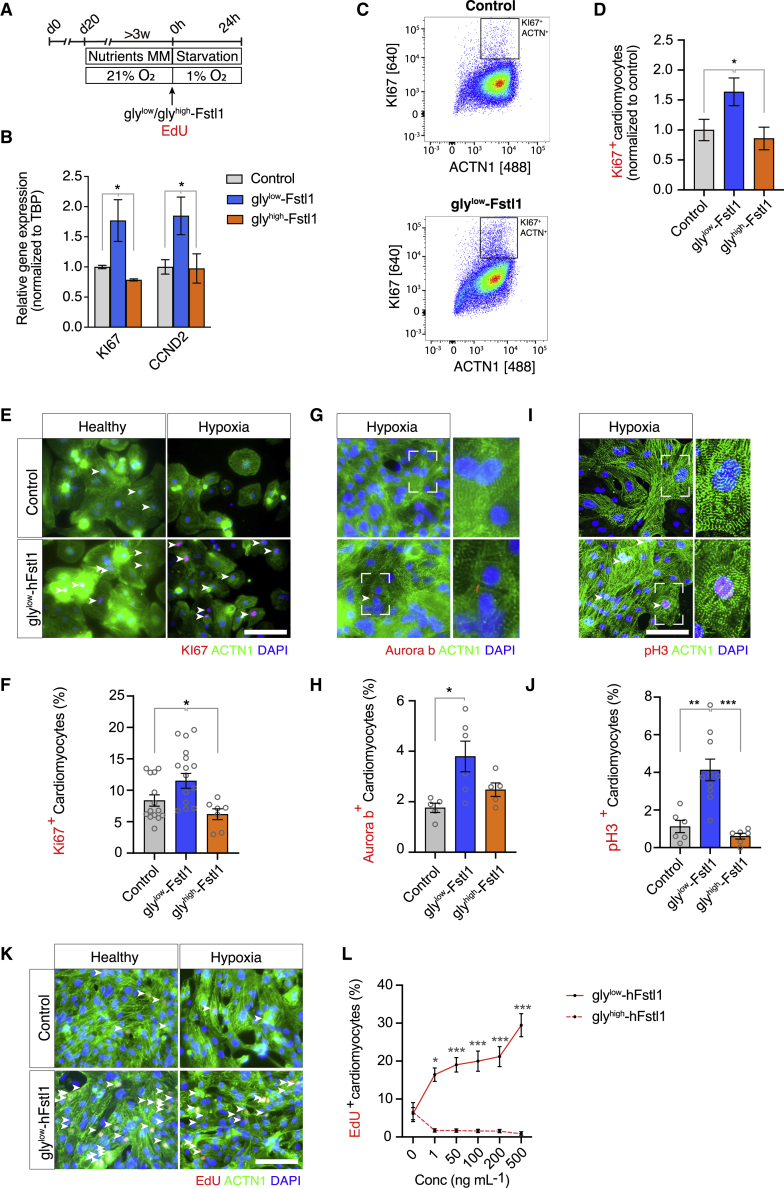
Gly^low^-FSTL1 treatment induces proliferation in normoxic and hypoxic matured iPSC-CMs (A) Schematic representation of experimental setup. (B) mRNA expression of cell cycle genes KI67 and CNND2 (CYCLIN D2) normalized to TATA binding protein (TBP) expression. (C and D) Flow cytometry analysis of KI67-positive iPSC-CMs (C) and quantification (D). (E–J) Representative images and quantification of KI67 (E and F), pH3 (G and H), and Aurora B kinase (I and J). (K and L) Microscopic images of EdU, ACTN1 staining (K), and quantification (L). Arrowheads indicate EdU + iPSC-CMs. n = 3 for staining and n = 6 for flow cytometry (both biological replicates). Data were analyzed using one-way ANOVA and Dunnett multiple-comparisons test. ∗p < 0.05, ∗∗p < 0.01, and ∗∗∗p < 0.001. Scale bar: 200 μm. Data are presented as mean ± SEM. To see this figure in color, go online.

To determine whether FSTL1 could promote proliferation in metabolically maturated iPSC-CMs, we exposed the cells to FSTL1 at the onset of 24 h hypoxia (Figure 2A). Gly^low^-FSTL1 treatment prior to 24 h hypoxia increased the mRNA expression of proliferation marker KI67 compared with control and G1/S cell cycle marker CYCLIN D2 compared with control (Figure 2B). Expression of proliferation marker KI67 was increased after gly^low^-FSTL1 treatment, as confirmed by flow cytometry (Figures 2C and 2D) and immunocytochemistry (Figures 2E and 2F), while treatment with gly^high^-FSTL1 did not increase KI67 expression (Figures 2D and S1). Similarly, only gly^low^-FSTL1 treatment increased expression of the cytokinesis marker Aurora B kinase (from 1.76% ± 0.18% [control] to 3.8% ± 0.61% [gly^low^-FSTL1]), p < 0.05; Figures 2G and 2H) and the mitotic marker pH3 (from 1.12% ± 0.33% [control] to 4.13% ± 0.57% [gly^low^-FSTL1), p < 0.01; Figures 2I and 2J).To further validate if FSTL1 increases proliferation, iPSC-CMs were incubated with 5-ethynyl-20-deoxyuridine (EdU) at the moment of FSTL1 administration, at the onset of 24 h hypoxia, to analyze DNA synthesis. gly^low^-FSTL1 dose-dependently increased EdU incorporation in hypoxic CMs, while gly^high^-FSTL1 did not (6.13 ± 1.6 [0 ng/mL gly^low^-FSTL1], 19.0% ± 1.91% [50 ng/mL gly^low^-FSTL1], and 1.69% ± 0.43% [50 ng/mL gly^high^-FSTL1]; Figures 2K and 2L).

**Figure 3:**
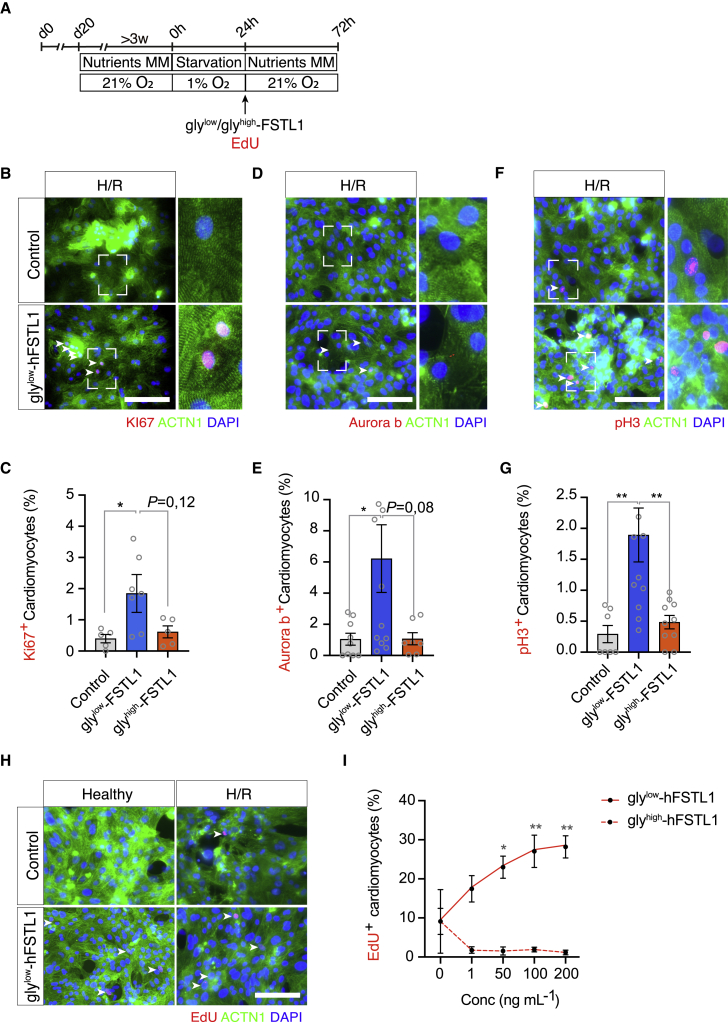
Gly^low^-FSTL1 treatment after hypoxia induces proliferation of iPSC-CMs (A) Schematic representation of experimental setup. (B–G) Representative images and quantification of KI67 (B and C), pH3 (D and E), and Aurora B kinase (F and G) after hypoxia and reoxygenation (H/R). (H and I) Microscopic images of EdU, ACTN1 staining (H), and quantification (I). Arrowheads indicate EdU + cardiomyocytes. n = 3 (biological replicates). Data were analyzed using one-way ANOVA and Dunnett multiple-comparisons test. ∗p < 0.05, ∗∗p < 0.01, and ∗∗∗p < 0.001. Scale bar: 200 μm. Data are presented as mean ± SEM. To see this figure in color, go online.

We have shown that gly^low^-FSTL1 induced proliferation of iPSC-CMs when supplemented at the start of hypoxia. To assess potential FSTL1-related effects on already damaged cells, we supplemented iPSC-CMs with FSTL1 24 h after hypoxia and analyzed for expression of proliferation markers after another 48 h of normoxic conditions (Figure 3A). Again, gly^low^-FSTL1 induced increased expression of KI67 (from 0.4% ± 0.13% [control] to 1.84% ± 0.61% [gly^low^-FSTL1), p < 0.05), pH3 (from 0.29% ± 0.14% [control] to 1.89% ± 0.44% [gly^low^-FSTL1]), p < 0.01), and Aurora B kinase (from 1.0% ± 0.38% [control] to 6.2% ± 2.2% [gly^low^-FSTL1]), p < 0.05) (Figures 3B–3G) compared with control. Furthermore, incorporation of EdU was significantly increased after treatment with gly^low^-FSTL1 compared with control (9.11 ± 3.3 [control] and 23.0% ± 2.84% [50 ng/mL gly^low^-FSTL1]; Figures 3H and 3I).

### RNA expression profile of FSTL1-treated hypoxic mature iPSC-CMs

**Figure 4:**
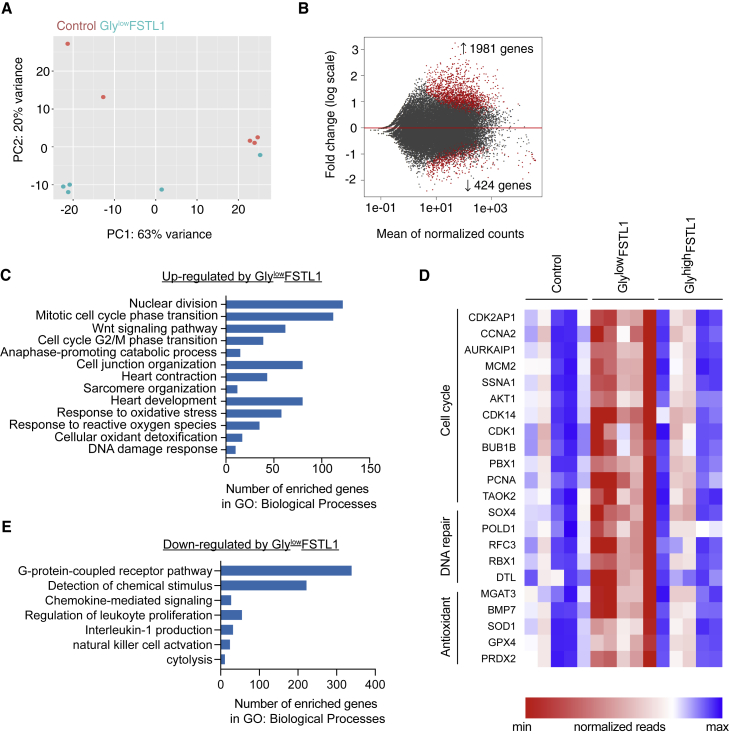
RNA expression profile of FSTL1-treated hypoxic iPSC-CMs (A) Principal component analysis (PCA) plots of hypo-glycosylated FSTL1-treated hypoxic iPSC-CMs and control hypoxic iPSC-CMs showing the separation between the samples on the basis of the top differentially expressed genes using DESeq2. (B) MA density plot of up- and down-regulated genes in gly^low^-FSTL1-treated iPSC-CMs compared with control using DESeq2. The x axis shows mean values of normalized counts of all samples, and the y axis shows log_2_ fold change in expression. (C) Gene Ontology (GO) enrichment analysis of GO term biological processes showing the top up-regulated biological processes enriched after treatment with gly^low^-FSTL1 compared with control. (D) Heatmap of normalized reads of significantly up-regulated cell cycle, DNA repair, or antioxidant genes after gly^low^-FSTL1 treatment compared with control, analyzed using Limma-voom differential expression analysis. Fold change compared with control is shown. (E) Gene Ontology enrichment analysis of GO term biological processes showing the top down-regulated biological processes enriched after treatment with gly^low^-FSTL1 compared with control. To see this figure in color, go online.

To assess the effect of the FSTL1 glycosylation variants on gene expression, we compared RNA sequencing (RNA-seq) transcriptional profiles of iPSC-CM after hypoxia and pre-treatment with gly^low^-FSTL1-, gly^high^-FSTL1-, and sham-treated controls (n = 5). Using DESeq2 analysis and an adjusted p value threshold of 0.1, we identified 2,405 differentially regulated genes with 1,981 up-regulated and 424 down-regulated genes in gly^low^-FSTL1-treated hypoxic iPSC-CMs compared with control hypoxic iPSC-CMs (Figures 4A and 4B). Principal component (PC) analysis showed scattering of the samples according to the largest variance between samples, indicating clustering on the basis of iPSC-CM cell line and experimental batch. No significantly differentially expressed genes could be found in gly^high^-FSTL1-treated hypoxic iPSC-CMs compared with control hypoxic iPSC-CMs (Figure S2). Gene Ontology enrichment analysis indicated that biological processes highly up-regulated in gly^low^-FSTL1-treated hypoxic iPSC-CMs were linked to cell proliferation (e.g., nuclear division, mitosis, cell junction organization), cardiac development and function, and cellular response to oxidative stress (Figure 4C). Analysis of specifically up-regulated genes after gly^low^-FSTL1 treatment showed up-regulation of cell cycle genes, DNA repair genes, and antioxidant genes, while treatment with gly^high^-FSTL1 did not induce similar up-regulation (Figure 4D). Among down-regulated biological processes after gly^low^-FSTL1 treatment were pathways linked to G protein-coupled receptor and immune system activation (Figure 4E). Differential expression analysis between control, gly^low^-FSTL1, and gly^high^-FSTL1 indicated significantly increased mRNA expression of cell cycle genes, DNA damage repair genes, and antioxidants after gly^low^-FSTL1 treatment. All together, we have shown that only gly^low^-FSTL1 specifically activates cell proliferation and reparative response to oxidative stress.

### Human cardiac fibroblasts secrete FSTL1, and co-culture with cardiomyocytes increases FSTL1 secretion

**Figure 5:**
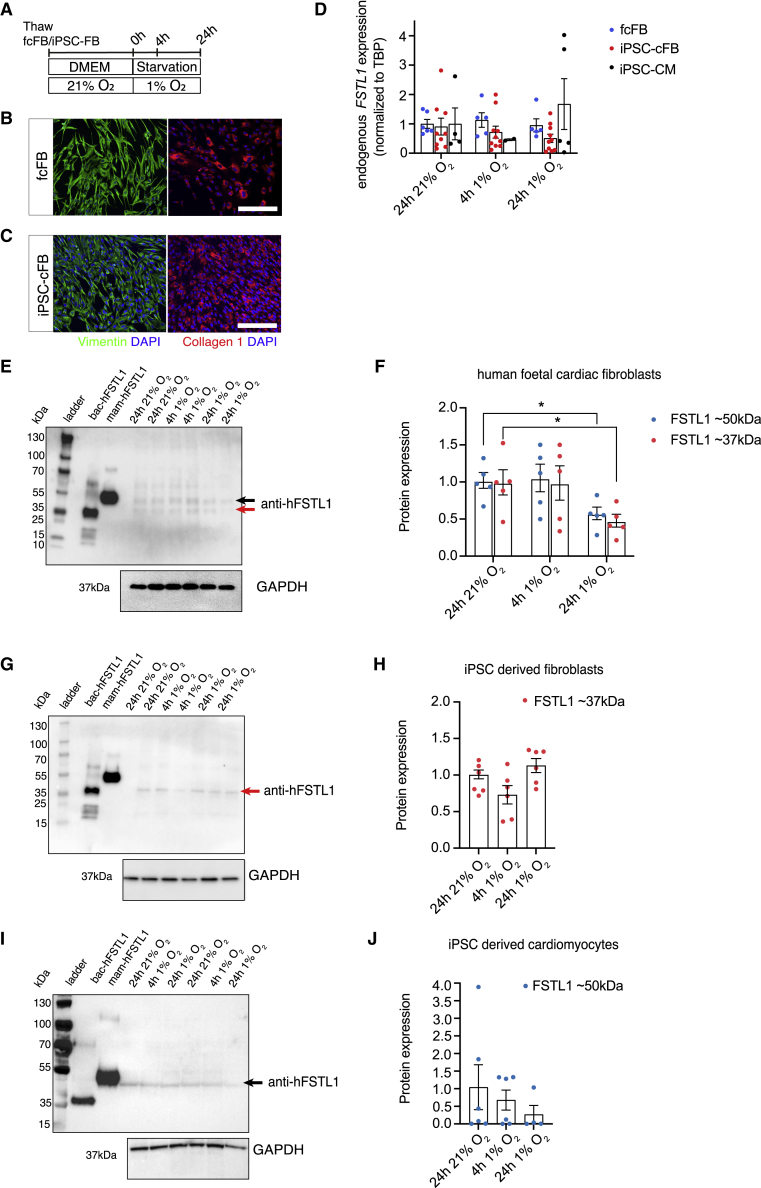
Normoxic and hypoxic human cardiac fibroblasts produce FSTL1 (A) Schematic representation of experimental setup. (B and C) Microscopic images of vimentin-1 and collagen 1 staining of human fetal cardiac fibroblasts (fcFBs) (B) and human iPSC-cFBs (C). (D) Endogenous FSTL1 mRNA expression levels normalized to TATA binding protein (TBP) levels. (E–J) FSTL1 protein expression levels of fcFBs (E and F), iPSC-cFBs (G and H), and iPSC-CMs (I and J) under normoxic and hypoxic conditions as determined using western blot. n = 3 (biological replicates). Data were analyzed using one-way ANOVA and Dunnett multiple-comparisons test. ∗p < 0.05, ∗∗p < 0.01, and ∗∗∗p < 0.001. Scale bar: 200 μm. Data are presented as mean ± SEM. To see this figure in color, go online.

We have shown that exposure to gly^low^-FSTL1 can induce protective effects and proliferation in hypoxia-challenged iPSC-CMs. As cFBs are present in the human heart at a similar frequency as cardiomyocytes^36^ and therefore could be a source of paracrine FSTL1 *in vivo*, we assessed FSTL1 expression of FSTL1 in fcFBs and iPSC derived cFBs (iPSC-cFBs; Figure 5A) in response to hypoxia. FcFBs and iPSC-cFBs expressed cell type-defining markers vimentin and collagen 1 (Figures 5B and 5C). qRT-PCR analysis for FSTL1 revealed all three cell lines, iPSC-CMs, and both cFBs, expressed at similar levels at normoxic conditions (Figure 5D). Change to hypoxic conditions showed no significant changes in mRNA expression in fcFBs, iPSC-cFBs, or iPSC-CMs. Analysis of intracellular (i.e., not secreted) FSTL1 protein expression showed that fcFBs and iPSC-cFB express FSTL1 protein at similar levels. Interestingly, fcFBs expressed FSTL1 of both molecular weights (∼37 and ∼50 kDa), presumably representing both glycosylation variants (Figures 5E and 5F), while iPSC-cFBs expressed only the lower molecular weight variant, with similar gel migration as the non-glycosylated FSTL1 (gly^low^) (Figures 5G and 5H). Hypoxic conditions decreased expression of both variants in fcFBs (Figures 5E and 5F), while no significant changes were observed in iPSC-cFBs (Figures 5G and 5H). Preliminary analysis of FSTL1 expression in iPSC-CMs showed predominant expression of high-molecular weight FSTL1 and a trend toward decreased expression over time of hypoxia exposure (Figures 5I and 5J).

**Figure 6:**
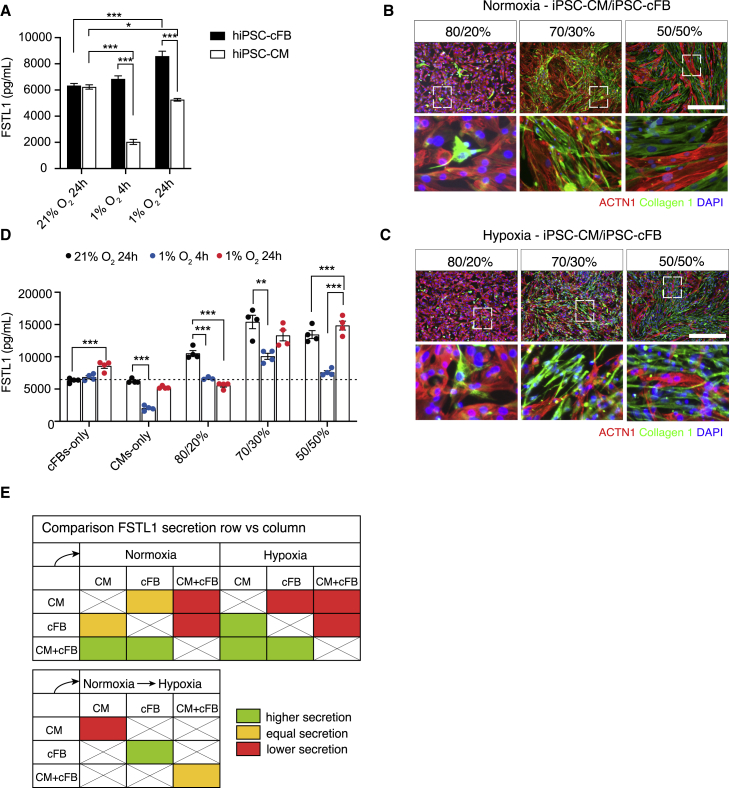
FSTL1 secretion is increased from iPSC-cFBs but decreased from iPSC-CMs in hypoxic conditions (A) FSTL1 medium concentrations as determined using Luminex assay. (B and C) Microscopic images of collagen 1 and α-actinin staining of cardiomyocyte/fibroblast co-cultures in normoxia (B) and hypoxia (C). (D) FSTL1 media concentrations in normoxia, short-term hypoxia, and long-term hypoxia as determined using Luminex assay. (E) Comparison of FSTL1 secretions of different cell types in normoxic and hypoxic conditions. Changes depict higher, equal, or lower expression of the cell types in the rows compared with the columns. n = 4 (biological replicates). Data were analyzed using one-way ANOVA and Dunnett multiple-comparisons test. ∗p < 0.05, ∗∗p < 0.01, and ∗∗∗p < 0.001. Scale bar: 200 μm. Data presented as mean ± SEM. To see this figure in color, go online.

We have shown that both fcFBs and iPSC-cFBs express FSTL1, and expression is decreased in prolonged hypoxia. We next aimed to determine the secretion of FSTL1 by iPSC-cFBs and iPSC-CMs and if secretion is changed by hypoxia. Media concentrations of secreted FSTL1 were determined with the Luminex multiplex detection system at normoxic baseline and after 4 or 24 h of hypoxia. In normoxic conditions, iPSC-CMs and iPSC-cFBs secreted similar levels of FSTL1 (6,327 ± 170.4 pg/mL [iPSC-cFBs] and 6,229 ± 163.1 pg/mL [iPSC-CMs]; Figure 6A). Interestingly, although hypoxia did not significantly increase internal FSTL1 protein levels in iPSC-cFBs, media FSTL1 levels increased in hypoxia (from 6,327 ± 170.4 pg/mL [24 h, 21% O_2_] to 8,582 ± 376.8 pg/mL [24 h, 1% O_2_]; p < 0.01). In iPSC-CMs, hypoxia decreased FSTL1 protein secretion (from 6,229 ± 163.1 pg/mL [24 h, 21% O_2_] to 5,250 ± 102.5 pg/mL [24 h, 1% O_2_], p < 0.01) (Figure 6A).

To determine whether FSTL1 secretion changes when both cell types are present, iPSC-CMs and iPSC-cFBs from the same cell line were co-cultured at 80%:20%, 70%:30% and 50%:50% ratios in normoxia or hypoxia. Immunofluorescence staining (iPSC-CMs: ACTN1; iPSC-cFBs: collagen 1) was used to visually confirm cell frequencies and distribution after seeding (Figures 6B and 6C). Co-culture of iPSC-CMs and iPSC-cFBs increased FSTL1 secretion levels in normoxic conditions (Figures 6D and S3A). Under hypoxic conditions, co-culture also increased FSTL1 secretion compared with mono-culture of iPSC-CMs and iPSC-cFB. However, for iPSC-CM/iPSC-cFB co-culture, hypoxia decreased FSTL1 secretion compared with normoxia, with the exception of 50%:50% iPSC-CM/iPSC-cFB co-culture, with increased FSTL1 secretion after long-term hypoxia (13,445 ± 613 pg/mL [21% O_2_] vs. 14,835 ± 688 pg/mL [24 h, 1% O_2_]; p < 0.001). Compared with short-term hypoxia (4 h, 1% O_2_), long-term hypoxia (24 h, 1% O_2_) increases FSTL1 secretion in mono-cultures and co-culture, except for a marked decrease of FSTL1 secretion in 80%:20% iPSC-CM/iPSC-cFB co-culture. Even though co-culture conditions showed decreased FSTL1 secretion after exposure to hypoxic conditions, FSTL1 secretion was still higher than in mono-cultures under the same conditions (with the exception of 80%:20% co-culture for short-term hypoxia, compared with iPSC-CM mono-culture).

Altogether, we reported marked differences in FSTL1 secretion between cell types, during co-culture of CMs and FBs and upon change from normoxic to long- and short-term hypoxia (Figures 6E and S3), pointing to the *in vivo* role of FSTL1 in CM-FB communication during normoxia and hypoxia, as present in ischemic heart disease. A shift in FSTL1 secretion from short- to long-term hypoxia could play a role in the post-hypoxic cardiac response.

## Discussion

In this study, we report that FSTL1 can protect human iPSC-CMs from hypoxia-induced cell death independent of protein glycosylation, and we found that non-glycosylated FSTL1 stimulates the proliferation of metabolically matured human CMs when administered either before or after injury. Furthermore, we report that both human iPSC-CMs and iPSC-cFBs secrete FSTL1, which upon change to hypoxic conditions decreases for iPSC-CMs but increases for iPSC-cFBs. Previously, iPSC-CMs have been reported to have an immature metabolic phenotype, relying mainly on anaerobic glycolysis for ATP production instead of oxidative phosphorylation, as seen in adult CMs.^37^, ^38^, ^39^ The metabolic demand affects the ability to reflect the phenotype of adult CMs during ischemic injury, as in the myocardium the absence of oxygen and nutrients induces a shift toward glycolytic metabolism.^40^, ^41^, ^42^ In this study, we used maturation media^35^ to shift the metabolism of the iPSC-CMs toward oxidative phosphorylation and therefore increase susceptibility to hypoxic conditions. As these metabolically matured human iPSC-CMs with very limited basal cell cycle activity could be stimulated by FSTL1 to proliferate upon exposure to hypoxic conditions, activation of CM proliferation in the adult human heart, seems possible but obviously needs further evaluation along the translational axis.

Previous studies have attempted to stimulate CM proliferation by targeting YAP1-Hippo,^43^, ^44^, ^45^ ErbB2,^46^^,^^47^ and Notch signaling,^48^^,^^49^ but so far, clinical translation has failed. The commonly used proliferation marker KI67 has been found to also be expressed in non-proliferating cells when there is overexpression of cell damage markers P53 and P21,^50^^,^^51^ which could bias reported effects of regenerative factors. However, the reduced regenerative effect in translational studies could also be related to differences between animal models and the human heart^52^^,^^53^ and delivery issues.^54^^,^^55^

Furthermore, with regenerative approaches the focus often is on the stimulation of CM proliferation, while restoration of vascularization and preventing extensive fibrosis are also required to enable cardiac regeneration. In light of this, studying regenerative factors that can also stimulate angiogenesis and target fibroblast behavior and function in intercellular communication could be a promising approach. We report that FSTL1 can double or triple the expression of multiple proliferation markers in human CMs to similar percentages of EdU and pH3 expression as reported in previous animal studies^44^^,^^56^ and higher Aurora B kinase levels than in previous studies.^43^^,^^46^ This, together with previous reports of FSTL1-mediated increase cardiac vascularization and cardioprotection, makes it a promising candidate factor to target cardiac repair. Additionally, we report differential gene expression after gly^low^-FSTL1 treatment in which all biological processes of cell proliferation (nuclear division, G2/M progression, mitosis, anaphase) and the protective response to oxidative stress and reactive oxygen species are up-regulated. Furthermore, genes involved in cytolysis and immune cell activation were significantly down-regulated after gly^low^-FSTL1 treatment, suggesting cardioprotective effects by modulating the pro-inflammatory activity of the immune system in the ischemic infarct. By decreasing both cell death and secretion of pro-inflammatory cytokines, the regenerative potential of FSTL1 might also be linked to increasing CM proliferation and survival in a less hostile (i.e., less inflammatory) microenvironment of the infarct.

The secretion of cardiokines plays important roles in intercellular communication during physiology and disease both by mediating paracrine cross-talk inside the heart and by facilitating communication with peripheral organs.^57^ To illustrate, the release of atrial natriuretic peptide (ANP) and brain natriuretic peptide (BNP) has been found to enable dynamic motion of the ventricular wall, while cardiokine IL-33 released by cFBs regulated CM hypertrophy via CM soluble protein ST2.^19^ Hypoxia-inducible cardiokine secretion has been reported in the murine heart, among which FSTL1 was identified as a cardioprotective cardiokine capable of preventing CM apoptosis.^16^ Cellular FSTL1 secretion by epicardial cells,^20^ CMs,^20^^,^^33^ and FBs^32^^,^^33^ has been reported in previous studies. In murine ischemic hearts, expression of FSTL1 was found to be essential in preventing cardiac rupture when expressed by cFBs,^32^ and expression of non-glycosylated FSTL1 was found to be capable of stimulating immature CMs to proliferate.^20^^,^^23^^,^^31^ In the human heart, elevated FSTL1 plasma levels and CM FSTL1 secretion have been reported in patients with heart failure.^57^, ^58^, ^59^, ^60^ As hypoxia impairs endoplasmic reticulum (ER) protein synthesis, protein secretion is decreased for most proteins.^16^^,^^61^ Maintained or increased protein secretion during ER stress suggests important functions for these proteins in the post-ischemic repair response.^16^ Potential mechanistic pathways of secreted FSTL1-mediated induction of cell survival and proliferation have been described^27^^,^^62^ to be linked to the DIP2A/PI3K/AKT1 axis,^63^ but further elucidation on (cardiac cell type) specific receptors and downstream targets is still required to fully understand the complex inter- and intracellular processes.

Our data demonstrate that hypoxic conditions induce an increase of FSTL1 secretion only from cardiac fibroblasts, while it decreases from iPSC-CMs. Furthermore, we found cFB cell line differences in FSTL1 expression, with fcFBs expressing both glycosylation variants, while iPSC-cFBs expressed only gly^low^-FSTL1. Intriguingly, FSTL1 expression in iPSC-CMs was shown to be exclusively gly^high^-FSTL1. It will be import to include analysis of glycosylation of secreted and thus paracrine-active FSTL1 in future studies to better understand this aspect of the complex intercellular communications taking place. Glycosylation has been linked before to biological activity of FSTL1 signaling.^20^^,^^31^ Differential expression of FSTL1 glycosylation variants during differentiation has been reported in pre-adipocytes to be associated with ability of thermogenesis.^64^ As FSTL1 has been found to be important for multiple processes during cardiac development and late myocardial cell formation,^62^^,^^65^ differential FSTL1 glycosylation might be enable beneficial to execute multiple functions, while late in development, predominant expression of gly^low^-FSTL1 by adult cardiac FBs might be more beneficial to mediate cardiac repair. As we show that only gly^low^-FSTL1 can induce CM proliferation, it seems likely that insufficient amounts of gly^low^-FSTL1 are linked to the limited innate reparative capacity of the heart. However, the mechanism and dynamics of intracellular and secreted FSTL1 during hypoxia remain to be elucidated. Interestingly, a recent study indicated a more pronounced role of cFBs, as opposed to epicardial cells,^22^ in the context of cardiac post-ischemic expression and activity of FSTL1. Furthermore, expression of FSTL1 by cFBs was reported to be associated with cFB migration and proliferation in murine infarcts as part of the acute cardiac repair response.^33^ Furthermore, higher levels of circulating FSTL1 in dogs were reported to prevent metabolic alterations in the failing heart and improve cardiac function.^66^ The increased presence of cFBs in the infarcted myocardium and expression of FSTL1 by cFBs in preventing cardiac rupture^32^ suggests a similar role of cFBs in FSTL1-mediated interplay with CMs in the ischemic myocardium. However, this potential induction of regenerative CM proliferation seems to be insufficient in mammalian hearts to effectively counteract cardiac damage in ischemic heart disease.

This is the first report of FSTL1 expression and secretion by human iPSC-cFBs and iPSC-CMs. We have shown that co-culture of both cell types increases FSTL1 secretion, indicating intercellular communication related to FSTL1. Interestingly, FSTL1 secretion from co-cultures under hypoxia was generally higher than from mono-cultures, while exposure to hypoxia mostly decreased FSTL1 secretion in iPSC-CM/iPSC-cFB co-cultures compared with normoxia. The complex nature of FSTL1 expression levels by iPSC-CMs and iPSC-FBs in co-culture between hypoxic and normoxic conditions requires further investigation. Further elucidation of FSTL1 signaling between CMs and cFBs in the healthy and ischemic heart could provide insights into how we can tap into this intercellular communication to support cardiac repair. As we confirm that FSTL1 acts as a secreted, paracrine factor, clinical translation could be accomplished via various delivery routes to ensure effective local concentrations, for example, by applying an epicardial patch,^20^ drug-releasing carriers such as injectable gels,^67^ or implantable mini-pumps.^68^ Large animal models using clinically applicable methodology^69^ seem an appropriate means to further investigate the translational potential of FSTL1 for ischemic heart disease.

## Materials and methods

### Cell culture

Human iPSC lines were kindly provided by Joseph Wu (SCVI-273) and Tomo Saric (NP0141-31B), and isolation of peripheral blood mononuclear cells and reprogramming were performed as previously described.^70^^,^^71^ iPSCs were grown in Essential 8 media (A1517001; Gibco) until they reached 90%–100% confluency. Differentiation to iPSC-CMs was initiated by changing media to RPMI 1640 (11875085; Thermo Fisher Scientific) and B27 minus insulin supplement (A1895601; Thermo Fisher Scientific) containing 7 μM CHIR99021 (S2924; Selleck Chemicals). After 3 days, canonical Wnt signaling was inhibited through the supplementation with Wnt-C59 (5148; R&D Systems). At day 7, the media was changed to RPMI 1640 and B27 plus insulin supplement (17504001; Thermo Fisher Scientific), after which the iPSC-CMs were purified in RPMI 1640 with no glucose (118979020; Thermo Fisher Scientific) at day 9. At day 11 of differentiation, cells were re-plated in 10% KnockOut Serum Replacement (KOSR) (108280028; Thermo Fisher Scientific) RPMI/B27 plus insulin. After a second purification step, media was changed to RPMI/B27 plus insulin. At day 20, media was changed to maturation media, as previously described.^35^ Cells were matured in maturation media for 3 weeks before re-plating.

iPSC-cFBs were differentiated from iPSCs as previously described.^72^ In short, differentiation was started at 100% confluency by changing media to RPMI 1640 and B27 minus insulin supplement containing 16 μM CHIR99021. After 2 days, media was changed to CFBM media supplemented with 75 ng/mL bFGF (WiCell Research Institute). Media was changed every other day with CFBM media^72^ supplemented with 75 ng/mL bFGF before passaging with TrypLE Express (12604013; Gibco) to DMEM (11995065; Gibco)/10% fetal bovine serum (12103C; Sigma Aldrich)/1% B27 plus insulin media at day 20.

Human fetal tissue was obtained following parental permission using standard informed consent procedures, and fcFBs were isolated as previously described.^73^

### Hypoxia simulation

Cells were exposed to 1% O_2_ in a hypoxia workstation (Baker Ruskinn InvivO_2_ 1000) or <1% O_2_ in a BD GasPak EZ Pouch system for 4 or 24 h after 24 h glucose starvation.

### FSTL1 supplementation

Recombinant human FSTL1 synthesized in *Escherichia coli* (gly^low^-FSTL1; Aviscera Bioscience) and in a mouse myeloma cell line (gly^high^-FSTL1; R&D Systems) were used.

Twenty-four hours prior to hypoxia or after hypoxia, cells were supplemented with FSTL1 (1–500 ng/mL) before measurement of EdU incorporation. Similarly, cells were supplemented with FSTL1 (100 ng/mL) for KI67, PH3, Aurora B kinase immunofluorescence staining, RNA expression analysis, and flow cytometric analysis.

### Immunofluorescence

Cells were fixed in 4% paraformaldehyde and permeabilized in 0.1% Triton X-100 before blocking in 10% normal goat serum/1% BSA (Millipore Sigma). Cells were incubated with primary antibodies (α-actinin [1:200; A7811; Sigma-Aldrich], cardiac troponin T [1:100; ab45932; Abcam], KI67 (1:200; ab8330; Abcam], PHH3 [1:200; 9701; Cell Signaling Technology], and Aurora B kinase [1:100; ab2254; Abcam]) overnight, and detection was mediated by incubation with secondary antibodies (Alexa Fluor antibody conjugates; Thermo Fisher Scientific) for 1 h. DAPI was used as a nuclear marker. Mounting was performed using Fluoromount-G mounting medium (Thermo Fisher Scientific).

To determine cell loss via pathways involving DNA fragmentation, TUNEL assays (Roche) were performed according to the manufacturer’s instructions. *In vitro* proliferation after FSTL1 supplementation was assessed using Click-iT EdU incorporation kit (Life Technologies). Imaging was performed using a confocal microscope (Leica SP8 X) and image analysis using ImageJ.

### RNA extraction and qPCR

RNA was extracted from the cells with TriPure isolation reagent (Roche) and reverse-transcribed to cDNA using the qScript cDNA synthesis kit (QuantaBio). Expression of target genes was determined using Perfecta SYBR Green SuperMix (QuantaBio) and gene-specific primers. TATA box binding protein (TBP) mRNA expression was used as a housekeeping reference, and expression levels were normalized to control using the ΔΔCT quantification method.

### RNA sequencing

After RNA extraction, RNA quality was determined using Qubit RNA HS Assay Kit (Thermo Fisher Scientific) and the Qubit fluorometer. The RNA library was prepared using the NEXTflex Rapid RNA-Seq Kit (Bio Scientific) and sequenced using CEL-seq (Illumina) to determine RNA expression. The Galaxy environment^74^ was used to analyze differential expression between two groups with DESeq2 and Limma-voom between more than two groups. Pairwise comparison between groups were conducted by applying the Wald test of the negative binomial distribution to the log_2_ gene counts, and significance was determined using an adjusted p value < 0.05. Because of the sensitive nature of the human RNA sequencing data collected for this study, requests to access the raw sequencing dataset from qualified researchers trained in human subject confidentiality protocols may be sent to the corresponding author.

### Western blotting

Protein extraction was mediated by RIPA lysis buffer (89901; Thermo Fisher Scientific) supplemented with phosphatase inhibitors (PhosSTOP; Roche) and protease inhibitors (cOmplete; Roche). After determining protein concentrations with Pierce BCA protein assay kit (Thermo Fisher Scientific), proteins were reduced using NuPAGE reducing agent (Thermo Fisher Scientific) and NuPAGE LDS sample buffer (Thermo Fisher Scientific) and loaded on 4%–15% acrylamide Mini-Protean TGX gel (Bio-Rad). After running gel electrophoreses, proteins were transferred to a PVDF membrane and blocked in 5% BSA in Tris-buffered saline with Tween (TBST). Primary antibody used was anti-FSTL1 (1:1,000; MAB1694; R&D Systems), and detection was mediated by secondary anti-rat horseradish peroxidase (HRP)-conjugated antibody (1:2,000; 31470; Thermo Fisher Scientific) in a Bio-Rad ChemiDoc Imager system.

### Flow cytometry

Cells were gently dissociated using the Multi Tissue Dissociation Kit (Miltenyi Biotec) and incubated using the LIVE/DEAD Fixable Green Dead cell stain kit (Thermo Fisher Scientific). Cells were fixed in inside fix solution (Miltenyi Biotec) and stained with primary antibodies diluted in inside perm solution (Miltenyi Biotec). Conjugated primary antibodies used were α-actinin-VioBlue (1:10; 130-106-996; Miltenyi Biotec) and KI67-APC (1:10; 130-111-761; Miltenyi Biotec). As a control, universal isotype control antibodies (REA; Miltenyi Biotec) were used. Media and washes were collected to obtain a complete representation of cell loss. The samples were analyzed using the FACS Canto system (BD Bioscience) and FlowJo software.

### Luminex bead-based array

Media were analyzed for FSTL1 protein levels using the Luminex magnetic bead-based multiplex assay (R&D Systems) according to the manufacturer’s instructions. In short, a standard curve was generated using calibrator diluent, and the standards and samples were transferred to a microplate. The microparticle cocktail was added to the wells, and the plate was incubated for 2 h at room temperature on a horizontal orbital microplate shaker, after which the plates were washed and consecutively incubated with the diluted Biotin-Antibody Cocktail and the diluted Streptavidin-PE cocktail. After resuspending the magnetic microparticles, the plates were read using a Luminex Analyzer (R&D Systems).

### Statistical analysis

Experiments were performed on iPSC lines from two donors. The n value is depicted in the figures for the individual experiments and represents the number of independent experiments performed with cells yielded from separate differentiations. Statistical analysis was performed using Prism 8 (GraphPad Software), and quantifications are represented as mean ± SEM. To compare the expression of 2 groups, a normality test was performed followed by Student’s t test. For >2 groups, one-way ANOVA was used after confirmation of normal distribution using the Shapiro-Wilk test. Post hoc analysis was done using Dunnett multiple-comparisons tests to determine statistically significant (p < 0.05) results.

### Study approval

Derivation and use of human iPSCs and human fetal cardiac progenitor cells or cFBs were approved by the ethics committee of University Medical Center Utrecht (Utrecht, the Netherlands). All subjects provided informed consent prior to participation.

## Supplemental information

Document S1. Figures S1–S3

Document S2. Article plus supplemental information
