# Supplementary material for: Follistatin-like 1 promotes proliferation of matured human hypoxic iPSC-cardiomyocytes and is secreted by cardiac fibroblasts
Source: Mol Ther Methods Clin Dev. 2022 Feb 23;25:3–16. doi: 10.1016/j.omtm.2022.02.005 (PMC8917270; doi:10.1016/j.omtm.2022.02.005)
Supplement: Document S1. Figures S1–S3 [file mmc1.pdf]

OMTM, Volume *25*

Supplemental information

Follistatin-like 1 promotes proliferation of matured human hypoxic iPSC-cardiomyocytes and is secreted by cardiac fibroblasts

Marijn C. Peters, Sofia Di Martino, Thomas Boelens, Jiabin Qin, Alain van Mil, Pieter A. Doevendans, Steven A.J. Chamuleau, Joost P.G. Sluijter, and Klaus Neef

Supplementary Information

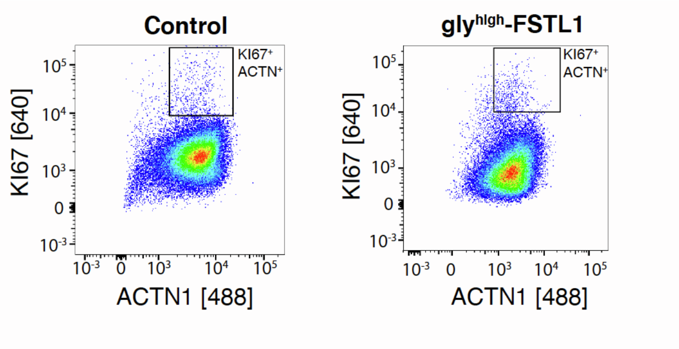

Supplementary figure 1. Gly^{high} FSTL1 does not increase iPSC-CM proliferation.

Flow cytometry analysis of KI67-positive iPSC-CMs treated with Gly^{high} FSTL1 compared to control.

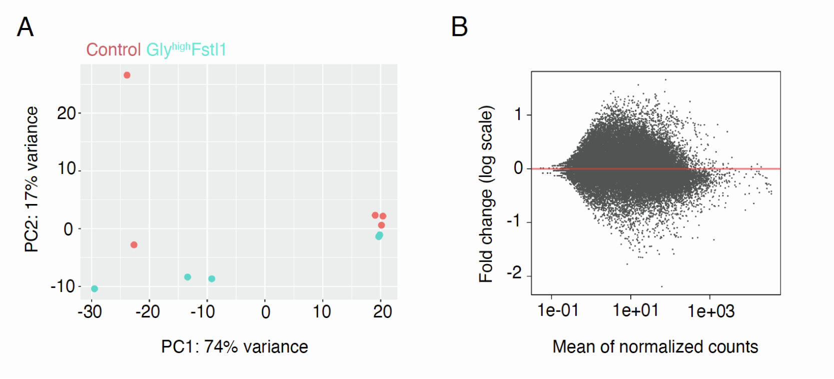

Supplementary figure 2. Treatment with gly^{high} FSTL1 does not induce differential gene expression.

A. Principle component analysis (PCA) plots of hyper-glycosylated FSTL1 treated hypoxic iPSC-CMs and control hypoxic iPSC-CMs showing the separation between the samples based on the top differentially expressed genes using DESeq2. **B**. MA density plot of differentially expressed genes in gly^{high}-FSTL1 treated iPSC-CMs compared to control using DESeq2. The x-axis shows mean values of normalized counts of all samples and the y-axis shows log2-fold change in expression.

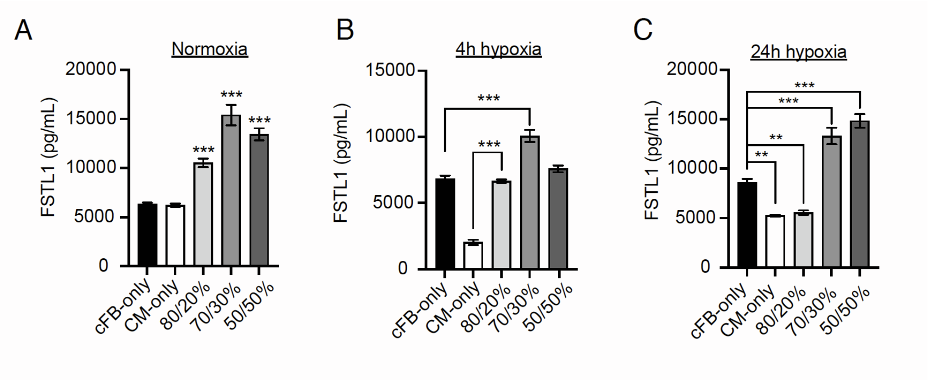

Supplementary figure 3. FSTL1 secretion profile in hypoxic iPSC-FB and iPSC-CM monoculture and coculture

A-C. FSTL1 concentration levels as determined by Luminex assay of conditioned media in normoxia (**A**), short term hypoxia (**B**), and long-term hypoxia (**C**). Data was analysed using one-way ANOVA and Dunnett multiple comparison. *P<0,05, ** P<0,01, ***P<0,001.
